# Supplementary material for: Grid-based computational methods for the design of constraint-based parsimonious chemical reaction networks to simulate metabolite production: GridProd
Source: BMC Bioinformatics. 2018 Sep 14;19:325. doi: 10.1186/s12859-018-2352-6 (PMC6137756; doi:10.1186/s12859-018-2352-6)
Supplement: Supplementary file 1 — All source codes and the solutions obtained by GridProd in the computational experiments described in this manuscript are included. (ZIP 2373 kb) [file 12859_2018_2352_MOESM1_ESM.zip › GridProdToolbox2.0/How to use GridProd.pdf]

How to use GridProd

About GridProd

GridProd calculates parsimonious metabolic networks for producing metabolites. GridProd divides the solution space of FBA into small grids, and conducts linear programming (LP) twice for each grid. The first LP obtains reactions included in the designed DNA, and the second LP calculates the production rate (PR) of the target metabolite under the condition that the growth rate (GR) is maximized for each grid. The design strategy of the grid whose PR is the best is then adopted as the GridProd solution.

Necessary environment

An environment where MATLAB and COBRA Toolbox work is required.

Estimation of computation time

The calculation times for the functions below were obtained on a genome-scale metabolic network of *E.coli*, iAF1260 (Feist *et al.* 2007), containing 1668 metabolites and 2382 reactions, with Gurobi, COBRA TOOLBOX and MATLAB on a Windows machine with Intel(R) Xeon(R) CPU E502630 v2 2.60GHz processors.
The average computation time of GridProd for a target metabolite in iAF1260 was 116 seconds in the default setting.

Run the test code for GridProd

The test code is run by the following command:

```
>> testGridProd()
```

"testGridProd" loads a MATLAB matfile containing a core metabolic model of *E. coli* (http://gcrg.ucsd.edu/Downloads/EcoliCore), and employs "GridProd", "BTconstraintSearch", "integrate", and "analyzeResult". It displays whether

the test was completed successfully or not. The calculation time is about 1 minute.

Example code
“example1”

```
>>load('iAF1260.mat')
% load the genome-scale COBRA model.

>> [targetProduction, minFlux, maxFlux, blockedRxns, usedRxns, biomass]
=GridProd(iAF1260,{'acser_e'},'EX_glc__D_e','EX_o2_e','BIOMASS_Ec_iAF1
260_core_59p81M');

% GridProd is employed.
% The target metabolite is "acser_e", which corresponds to "EX_acser(e)".
% The glucose reaction is 'EX_glc__D_e'.
% The oxygen reaction is 'EX_o2_e'.
% The biomass objective fucntion reaction is
% 'BIOMASS_Ec_iAF1260_core_59p81M'.

% Since the options are not specified,
% the glucose uptake ratio is 10,
% the oxygen uptake ratio is 5,
% the minimum growth ratio is 0.05 as defaults,
% the size of each grid is (TMGR/25)(TMPR/25).
%

>> minFlux
>> maxFlux

% 7.1031 is obtained as the minimum and maximum production rates for O-
Acetyl-L-serine production.
% The corresponding growth rate is 0.1496.

>> paintGrid('results/analyzeResult_339.mat')
```

% 'paintGrid' shows the heatmap that shows the levels of O-Acetyl-L-serine
%   production rate for each grid.
% Note that 339 is ID of O-Acetyl-L-serine in iAF1260.
save('example1.mat');

"example2"

>>load('ecoli_core_model.mat')
%   load the ecoli core model.

>> [targetProduction, minFlux, maxFlux, blockedRxns, usedRxns, biomass]=GridProd(model,{'succ[c]'},'EX_glc(e)','EX_o2(e)','Biomass_Ecoli_core_w_GAM', 'GUR',8,'OUR',18.5,'minGrowth',0.01,'P',10);

% GridProd is employed.
% The target metabolite is "succ[c]".
% The glucose reaction is 'EX_glc(e)'.
% The oxygen reaction is 'EX_o2(e)'.
% The biomass objective fucntion reaction is
% 'Biomass_Ecoli_core_w_GAM'.

% The options are specified as follows.
% the glucose uptake ratio is 8,
% the oxygen uptake ratio is 18.5,
% the minimum growth ratio is 0.01.
% P is 10
.
>> minFlux
>> maxFlux

% 9.9904 is obtained as the minimum and maximum production rates for succinate.

>> paintGrid('results/analyzeResult_69.mat')
%
% 'paintGrid' shows the heatmap that shows the levels of succinate

% production for each grid.
% Note that 69 is ID of succinate in the ecoli core model.

Perform GridProd

GridProd identifies a set of unused reactions for production of target metabolites, and is executed as follows.

```
>> [targetProduction, minFlux, maxFlux, blockedRxns, usedRxns, biomass]
= GridProd(model, targetMet,   glucoseRxn, oxygenRxn, biomassRxn,
options)
```

INPUTS

model	COBRA model structure containing the following required fields to perform GridProd.
rxns	Rxns in the model
mets	Metabolites in the model
S	Stoichiometric matrix (sparse)
b	RHS of Sv = b (usually zeros)
c	Objective coefficients
lb	Lower bounds for fluxes
ub	Upper bounds for fluxes
rev	Reversibility of fluxes
targetMet	target metabolites (e.g., {'succ_c','glu__D_c'})
glucoseRxn	Reaction representing glucose uptake (e.g., EX_glc__D_e)
oxygenRxn	Reaction representing oxygen uptake (e.g., EX_o2_e)
biomassRxn	Reaction representing biomass objective function (e.g., BIOMASS_Ec_iAF1260_core_59p81M)

OPTIONAL INPUTS

GUR Glucose uptake ratio (Default: 10)
OUR Oxygen uptake ratio (Default: 5)
minGrowth The minimum value of biomass objective function that the designed strain must achieve. (Default: 0.05)
P paramters for the grid size (described as P^{-1} in the manuscript, Default:25)

OUTPUTS
targetProduction The production rate of target metabolites achieved by the designed metabolic network.
minFlux The minimum values of the target metabolite production obtained by FVA.
maxFlux The maximum values of the target metabolite production obtained by FVA.
blockedRxns A set of unused reaction that achieves the value of targetProduction
usedRxns A set of reactions that is not included in blockedRxns
biomass The value of biomass objective function when blockedRxns is not used.

Perform PaintGrid

“paintGrid” outputs a heatmap that shows how much target metabolite production is achieved for each grid.

```
>> paintGrid(resultFile)
```

INPUTS
resultFile The name of the file should be “analyzeResult_%d.mat” where %d is ID in the model, and the file should be output by “analyzeResult”
OUTPUTS
The heatmap figure is shown.

Reference

Feist, A. M., Henry, C. S., Reed, J. L., Krummenacker, M., Joyce, A. R., Karp, P. D.,Broadbelt, L. J., Hatzimanikatis, V., and Palsson, B. Ø. (2007). A genome-scale metabolic reconstruction for escherichia coli k-12 mg1655 that accounts for 1260 orfs and thermodynamic information. Molecular systems biology, 3(1), 121.
